# Floquet Weyl Magnons in Three-Dimensional Quantum Magnets

**DOI:** 10.1038/s41598-018-28508-5

**Published:** 2018-07-04

**Authors:** S. A. Owerre

**Affiliations:** Perimeter Institute for Theoretical Physics, 31 Caroline St. N., Waterloo, Ontario, N2L 2Y5 Canada

## Abstract

In three-dimensional (3D) quantum magnets, magnonic Weyl points (WPs) featuring linear band crossing of two non-degenerate magnon branches can emerge in certain lattice geometry when time-reversal symmetry is broken macroscopically. Unfortunately, there are very limited 3D quantum magnets that host magnonic WPs, and they are yet to be observed experimentally because the intrinsic perturbative interactions that break time-reversal symmetry macroscopically can be very negligible. Here, we present an alternative means via photo-irradiation, in which magnonic WPs can emerge in 3D quantum magnets without relying on intrinsic perturbative interactions to break time-reversal symmetry. By utilizing the magnonic Floquet-Bloch theory, we put forward the general theory of magnonic Floquet WPs in 3D quantum magnets. We show that periodically driven 3D magnonic Dirac nodal-line (DNL) and 3D magnonic gapped trivial insulators can generate 3D magnonic Floquet WPs, which can be tuned by the incident circularly-polarized light. We demonstrate the existence of magnonic Floquet WPs by combining the study of the magnon dispersions, Berry curvatures, and the anomalous thermal Hall effect. The general theoretical formalism can be applied to different magnetic insulators, and thus extending the concept of magnonic WPs to a broader class of 3D magnetically ordered systems.

## Introduction

The condensed matter realization of Weyl semimetals as emergent quasiparticles hosting Weyl fermions has attracted considerable interest in recent years^1–4^. Weyl semimetals are considered to be the first material realization of Weyl fermions in nature. Generically, WPs are allowed in 3D solid-state materials with either broken inversion (${\mathscr{T}}$) or time-reversal (${\mathscr{T}}$) symmetry. This guarantees that two WPs separated in momentum space are topologically stable and can only disappear by pair annihilation^5,6^. Essentially, the general notion of WPs in condensed-matter systems is manifested when two non-degenerate topologically protected bands cross linearly in 3D momentum space. This linear band crossing point is independent of the quasiparticle excitations and their corresponding quantum statistics. Therefore, it occurs in both bosonic and fermionic systems. Recently, magnonic WPs have come into focus^7–14^ as the bosonic analogs of electronic WPs, and they occur in 3D (as well as quasi-2D) insulating ordered magnets when two non-degenerate magnon branches cross linearly in the 3D Brillouin zone (BZ).

In magnetic Weyl systems, ${\mathscr{T}}$-symmetry is naturally broken owing to the presence of magnetic order. Nonetheless, magnonic WPs generally do not exist in every 3D magnetic material. The existence of stable magnonic WPs can be achieved when ${\mathscr{T}}$-symmetry is macroscopically (explicitly) broken. For insulating quantum ferromagnets, macroscopically broken ${\mathscr{T}}$-symmetry can be achieved by the combination of spontaneous magnetization and the Dzyaloshinskii-Moriya (DM) interaction^15,16^ in the direction of the magnetization. The DM interaction is allowed in quantum magnets that lack an inversion center, and it plays the role of spin-orbit coupling (SOC). For insulating quantum antiferromagnets, however, the antiferromagnetic order can be restored by symmetry, hence the spontaneous magnetization and the DM interaction can be inadequate to provide stable magnonic WPs in antiferromagnets. In this case, one can only achieve magnonic WPs through symmetry-breaking noncoplanar spin textures with nonzero scalar spin chirality or applied external magnetic field. The former provides a possible transition to chiral spin liquids in which ${\mathscr{T}}$-symmetry is also broken macroscopically.

One of the hallmarks of Weyl semimetals is the appearance of the Fermi arc surface states, which connect the surface projection of WPs in momentum space^1,2^. This provides a distinct topological classification from topological insulators (TIs). Besides, WPs are also sinks and sources of the Berry curvature. In other words, a single WP acts as a (magnetic) monopole of the Berry curvature in momentum space. Similarly, magnonic WPs also host magnon arc surface states as a topological feature, and they are also the monopoles of the Berry curvature in momentum space. Despite the simplicity of the theoretical concepts of WPs in quantum materials, their experimental realizations in real materials are elusive. This is in part due to the fact that the intrinsic perturbative interactions that are necessary for WPs to occur can be very weak or the quantum materials may have strong correlated many-body effects. Thus far, the experimental realizations of bosonic WPs have only been reported in artificial photonic and phononic optical systems^17,18^. Therefore, it is desirable to explore other possibilities in which bosonic magnetic WPs can be witnessed in quantum materials.

In recent years, photo-irradiation of solid-state materials have emerged as an alternative means to extend the search for topological quantum materials^19^. By exposing a topologically trivial quantum material to a time-periodic electromagnetic (laser) field, the intrinsic properties of the material can be altered via light-matter interactions. Basically, the charge carriers in the quantum material couple to the time-periodic vector potential through a time-dependent Peierls phase, in a similar way to the Aharonov-Bohm phase^20^. Consequently, the quantum material becomes a periodically driven system, which can be studied by the Floquet-Bloch theory. The resulting effect of irradiated quantum materials is that ${\mathscr{T}}$-symmetry breaking terms can be photo-induced, leading to different nontrivial Floquet topological phases such as Floquet topological insulators^21–35^ and Floquet Weyl semimetals^36–44^.

In fact, the mechanism of photo-irradiation is not restricted to electronic charge materials, but also applies to solid-state materials with charge-neutral carriers. In particular, charge-neutral magnons are simply magnetic dipole moments hopping in an ordered magnetic insulator, and they produce a force similar to the Lorentz force on charged particles^45^. Therefore, magnons can also couple to a time-independent electric field through the Aharonov-Casher effect^46–48^–a mechanism in which charge-neutral particles acquire a geometric phase in an electric field background. In this formalism, magnonic Landau levels can be induced in insulating magnets^49^, and chiral anomaly can be induced in Weyl magnons^9,11^, in analogy to electronic systems. However, the physics of time-independent electric field is completely different from that of time-periodic electric field from a laser source. In the latter, one realizes a time-dependent version of the Aharonov-Casher effect (see Methods), which leads to periodically driven magnetic insulators also amenable to solution via the Floquet-Bloch theory. The magnonic Floquet-Bloch theory describes the interaction of light with magnonic Bloch states in insulating quantum magnets. Consequently, two-dimensional (2D) Dirac magnons in honeycomb ferromagnets can be driven to 2D magnonic Floquet TIs via a photoinduced next-nearest-neighbour DM interaction^50^, and also topological phase transition can be photoinduced in intrinsic magnon TIs such as Cu(1–3, bdc)^51^. Thus, magnonic systems can now be studied in analogy to photo-irradiated graphene and Chern insulators, which generate 2D electronic Floquet TIs^21,23,25^ and photoinduced topological phase transition^22^ respectively.

In this report, we generalize this new concept to 3D insulating quantum magnets. In this case the incident light can be applied in different directions due to the 3D nature of the system, but not all directions generate WPs (In contrast to undriven magnonic WPs induced by the *z*-component of the DM vector^8–11^, we will show that the *z*-component of the photoinduced DM interaction is not the source of the magnonic Floquet WPs in the current 3D ferromagnetic system). We will start with a 3D quantum magnets with Dirac nodal-line (DNL) phase in which the Dirac points (DPs) form closed loops in the BZ. By fine-tuning the model parameters the DNLs can be gapped out to a trivial insulator. Therefore, our 3D quantum magnet has two phases that are topologically trivial. Our main goal is to generate topologically nontrivial phase from this system by applying photo-irradiation in different directions of the 3D quantum magnet. In particular, we show that while photo-irradiation in all other direction generates 3D magnonic Floquet TIs, photo-irradiation in the direction parallel to the DNLs generates 3D magnonic Floquet WPs, which is very similar to electronic Floquet systems^39,40^. We also observe that tunable 3D magnonic Floquet WPs can emerge from periodically driven 3D magnonic gapped trivial insulator using circularly-polarized lights. We establish a compelling evidence of magnonic Floquet WPs in this 3D insulating quantum magnet by computing the monopole distributions of the Berry curvature in momentum space and the thermal Hall conductivity, both of which vanish in quantum magnets with ${\mathscr{T}}$-symmetry, such as the undriven Dirac magnons, or DNL magnons, or trivial magnon insulators. The theoretical formalism and the results are general, and can be applied to different magnetic insulators, including the recently observed Dirac magnons in 3D antiferromagnet Cu_3_ TeO_6_^52–54^. We envision that our results will greatly impact future research in magnonic topological systems, and extend the experimental search for magnonic WPs to a broader class of 3D quantum magnetic insulators, with potential practical applications to features such as photo-magnonics^55^, magnon spintronics^56,57^, and ultrafast optical control of magnetic spin currents^58–61^.

## Results

### Spin Model

**Figure 1:**
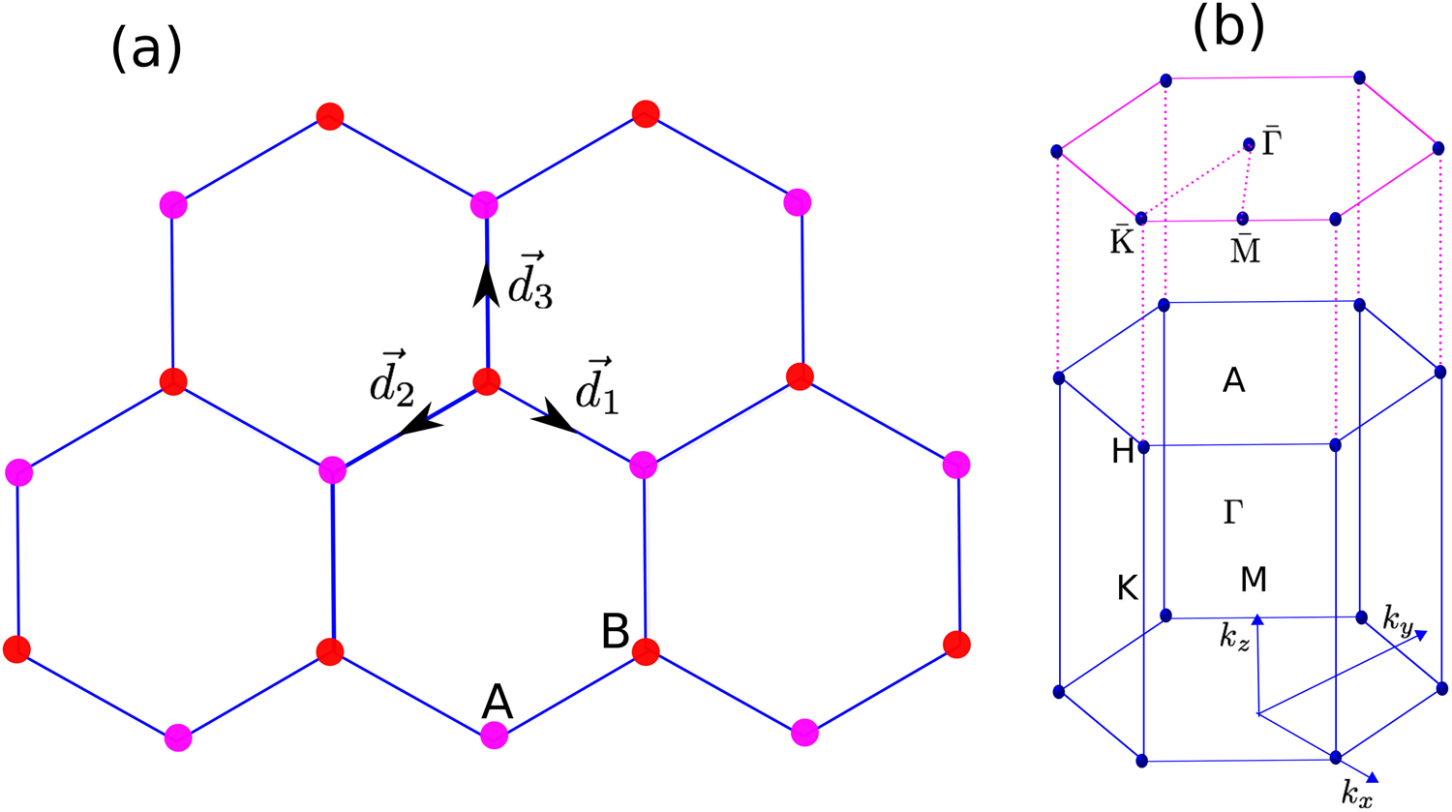
(**a**) Top view of the honeycomb ferromagnetic layers with vertical bond stacked along the (001) direction. (**b**) The bulk Brillouin zone (BZ) and its projection onto the hexagonal (001) surface BZ.

We study the simple Heisenberg spin Hamiltonian of layered ferromagnets, governed by

1 $$ {\mathcal H} =-\,J\sum _{\langle ij\rangle ,\ell }{\overrightarrow{S}}_{i,\ell }\cdot {\overrightarrow{S}}_{j,\ell }-{J}_{L}\sum _{\langle \ell \ell ^{\prime} \rangle ,i}{\overrightarrow{S}}_{i,\ell }\cdot {\overrightarrow{S}}_{i,\ell ^{\prime} },$$

where ${\overrightarrow{S}}_{\ell }=({S}_{\ell }^{x},{S}_{\ell }^{y},{S}_{\ell }^{z})$ is the spin vector at site $\ell$. Here *J* and *J*_*L*_ are the intralayer and interlayer (vertical bond) ferromagnetic interactions respectively. The Hamiltonian in Eq. 1 is applicable to different layered ferromagnets in various lattice geometries. In this report, we will focus on honeycomb layered ferromagnets. In Fig. 1(a,b) we have shown the top view of the honeycomb lattice stacked with a vertical bond along the (001) direction and its 3D Brillouin zone (BZ) respectively. Indeed, most realistic bulk layered honeycomb ferromagnetic materials such as the honeycomb chromium compounds CrX_3_ (X ≡ Br, Cl, and I)^62–66^, have an inversion center. Therefore, the DM interaction is forbidden by symmetry in these materials. We would like to mention that the realistic parameter regime of the spin Hamiltonian in Eq. (1) is not the main focus in this report. Our main objective is to demonstrate how magnonic Floquet WPs can be generated by periodic driving of 3D DNL magnons and 3D trivial magnon insulators, which are obtainable from Eq. (1) in different parameter regimes. In order to achieve this goal, we consider honeycomb ferromagnetic layers stacked similarly to ABC-stacked graphene^67–71^.

### Undriven magnonic Dirac nodal-line

The concept of DNLs emerges when the DPs form a loop or ring in the BZ. This usually happens in 3D systems without explicit ${\mathscr{T}}$-symmetry breaking terms or other forms of symmetry protection. In this section, we will introduce this concept using the underlying magnetic excitations of the spin Hamiltonian in Eq. (1). In the low temperature regime, the magnetic excitations of ordered ferromagnetic insulators are charge-neutral magnons, and they can be captured by the Holstein Primakoff (HP) transformation^72^: ${S}_{i,\ell }^{z}=S-{a}_{i,\ell }^{\dagger }{a}_{i,\ell },\,{S}_{i,\ell }^{+}\approx \sqrt{2S}{a}_{i,\ell }={({S}_{i,\ell }^{-})}^{\dagger }$, where ${a}_{i,\ell }^{\dagger }({a}_{i,\ell })$ are the bosonic creation (annihilation) operators, and ${S}_{i,\ell }^{\pm }={S}_{i,\ell }^{x}\pm i{S}_{i,\ell }^{y}$ denote the spin raising and lowering operators. The corresponding non-interacting magnon Hamiltonian is given by ${\mathcal H} ={\sum }_{\overrightarrow{k}}{\psi }_{\overrightarrow{k}}^{\dagger } {\mathcal H} (\overrightarrow{k}){\psi }_{\overrightarrow{k}}$ with ${\psi }_{\overrightarrow{k}}^{\dagger }=({a}_{\overrightarrow{k},A}^{\dagger },{a}_{\overrightarrow{k},B}^{\dagger })$,

2 $$ {\mathcal H} (\overrightarrow{k})={\rho }_{0}{1}_{2\times 2}+(\begin{array}{cc}0 & \rho (\overrightarrow{k})\\ {\rho }^{\ast }(\overrightarrow{k}) & 0\end{array}),$$

where 1_2×2_ is an identity matrix. *ρ*_0_ = 3*JS* + *J*_*L*_*S* and $\rho (\overrightarrow{k})=\rho ({\overrightarrow{k}}_{\parallel })+\rho ({k}_{z})$, with $\rho ({k}_{z})=-\,{t}_{L}\exp (i{k}_{z})$, $\rho ({\overrightarrow{k}}_{\parallel })=$ $-t{\sum }_{j}{e}^{i{\overrightarrow{k}}_{\parallel }\cdot {\overrightarrow{d}}_{j}}$. Here, *t*_*L*_ = *J*_*L*_*S*, *t* = *JS*, ${\overrightarrow{d}}_{1}=(\sqrt{3}/2,-1/2)$, ${\overrightarrow{d}}_{2}=-\,(\sqrt{3}/2,1/2)$, and ${\overrightarrow{d}}_{3}=(0,1)$. The total momentum vector is defined as $\overrightarrow{k}=({\overrightarrow{k}}_{\parallel },{k}_{z})$, where the in-plane wave vector is ${\overrightarrow{k}}_{\parallel }=({k}_{x},{k}_{y})$. Using the Pauli matrices *σ*_*i*_ (*i* = *x*, *y*, *z*), we write the Hamiltonian (2) as

3 $$ {\mathcal H} (\overrightarrow{k})={f}_{0}{\sigma }_{0}+{f}_{x}(\overrightarrow{k}){\sigma }_{x}+{f}_{y}(\overrightarrow{k}){\sigma }_{y},$$

where *σ*_0_ ≡ 1_2 × 2_ and *f*_0_ = *ρ*_0_,

4 $${f}_{x}(\overrightarrow{k})=-\,t\sum _{j}\cos ({\overrightarrow{k}}_{\parallel }\cdot {\overrightarrow{d}}_{j})-{t}_{L}\,\cos ({k}_{z}),$$

5 $${f}_{y}(\overrightarrow{k})=t\sum _{j}\sin ({\overrightarrow{k}}_{\parallel }\cdot {\overrightarrow{d}}_{j})+{t}_{L}\,\sin ({k}_{z}\mathrm{).}$$

The pseudospin time-reversal symmetry operator is ${\mathscr{T}}={\sigma }_{0}{\mathscr{K}}$, where ${\mathscr{K}}$ is complex conjugation. Evidently, the Hamiltonian in Eq. (3) is ${\mathscr{T}}$-invariant. The condition for DNLs to exist requires ${f}_{x}(\overrightarrow{k})={f}_{y}(\overrightarrow{k})=0$. This condition is satisfied in the *k*_*z*_ = *π* plane at *k*_*y*_ = 0 and ${k}_{x}=\pm \,{k}_{x}^{{\rm{D}}}$, where

6 $${k}_{x}^{{\rm{D}}}=\frac{2}{\sqrt{3}}\arccos (\frac{-1+{t}_{L}/t}{2}).$$

**Figure 2:**
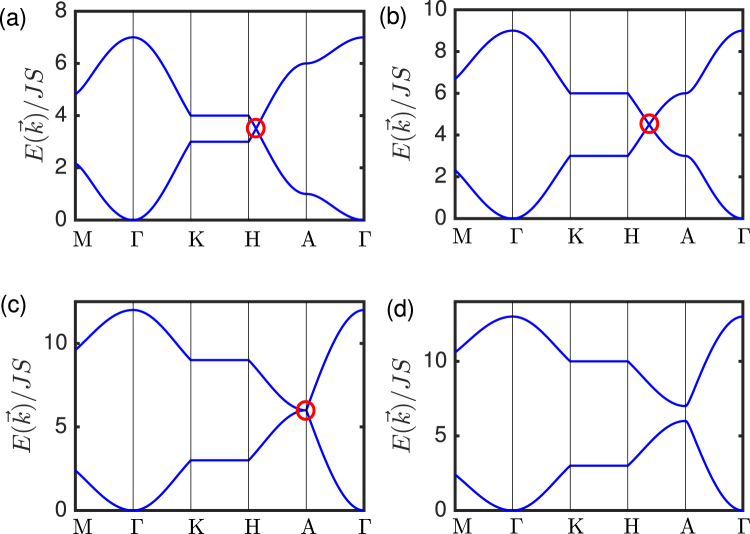
Evolution of the magnon bands of undriven layered honeycomb ferromagnets, showing the phase transition from 3D DNL magnons to 3D gapped trivial magnon insulator. (**a**) *t*_*L*_/*t* = 0.5, (**b**) *t*_*L*_/*t* = 1.5, (**c**) *t*_*L*_/*t* = 3, (**d**) *t*_*L*_/*t* = 3.5. The red circles denote the DNLs.

For ${t}_{L}/t < 3$, the DPs form loops or rings centred at the $\bar{{\rm{K}}}$-point in the (001) surface BZ, and thereby realize DNLs. For the Dirac nodal loops centred at the $\bar{{\rm{K}}}$-point in the *k*_*z*_ = *π* plane, the expression for the loops is ${q}_{x}^{2}+{q}_{y}^{2}={({t}_{L}/{v}_{s})}^{2}$, where *v*_*s*_ = 3*t*/2 is the group velocity, and $\overrightarrow{q}=\bar{{\rm{K}}}-{\overrightarrow{k}}_{\parallel }$ is the momentum deviation from the DNL. In the regime ${t}_{L}/t > 3$, a gapped trivial insulator is obtained. In this report, we will study both the DNLs and the gapped trivial insulator. The phase transition from DNLs to gapped trivial insulator is depicted in Fig. (2). Note that in the vicinity of the DNLs at the $\bar{{\rm{K}}}$-point, the functions ${f}_{x}(\overrightarrow{k})$ and ${f}_{y}(\overrightarrow{k})$ are linear in *k*_*x*_ and *k*_*y*_ respectively. Since ${\mathscr{T}}$-symmetry is preserved, the Berry curvature of the DNLs vanishes. Therefore, their topological protection is only characterized by the Berry phase defined as $\gamma ={\oint }_{{\mathscr{C}}}{\mathscr{A}}(\overrightarrow{k})\cdot d\overrightarrow{k}$, over a closed loop ${\mathscr{C}}$, where ${\mathscr{A}}(\overrightarrow{k})$ is the Berry connection given by ${\mathscr{A}}(\overrightarrow{k})=-\,i\langle {\psi }_{\overrightarrow{k}}^{\dagger }|{\overrightarrow{\nabla }}_{\overrightarrow{k}}{\psi }_{\overrightarrow{k}}\rangle$, and ${\psi }_{\overrightarrow{k}}$ is the magnon eigenvectors. For a closed path encircling the DNLs in momentum space, the Berry phase is *γ* = *π*, otherwise *γ* = 0.

### Driven magnonic Dirac nodal-line and Photoinduced Weyl magnons

The notion of periodically driven magnonic systems essentially rely on the quantum theory of magnon quasiparticles. Magnons are in fact moving magnetic dipoles in a magnetically ordered system and they are charge-neutral bosonic quasiparticle with an intrinsic spin-1. The magnetic dipole moment is given by $\overrightarrow{\mu }={\mu }_{m}\hat{z}$, where *μ*_*m*_ = *gμ*_*B*_, *g* is the spin *g*-factor and *μ*_*B*_ is the Bohr magneton. Therefore, magnons can couple to electric fields through the Aharonov-Casher effect^45–48^, similar to the coupling of charged particles through the Aharonov-Bohm effect^20^. In general, a neutral particle couples non-minimally to an external electromagnetic field (see Methods).

In the current study, we will apply this new concept to 3D insulating quantum ferromagnets possessing 3D DNL magnon and 3D gapped trivial magnon insulator phases. We consider photo-irradiation of magnons in the insulating quantum ferromagnets described by the pristine Hamiltonian in Eq. (1). In the case of time-periodic electromagnetic field possessing a dominant time-dependent electric field components $\overrightarrow{ {\mathcal E} }(\tau )$, the effects of the electric field can be described by a vector potential $\overrightarrow{{\mathscr{A}}}(\tau )$, where $\overrightarrow{ {\mathcal E} }(\tau )=-\,\partial \overrightarrow{{\mathscr{A}}}(\tau )/\partial \tau$. The time-periodicity guarantees that $\overrightarrow{{\mathscr{A}}}(\tau +T)=\overrightarrow{{\mathscr{A}}}(\tau )$, with *T* = 2*π*/*ω* being the period. In the real space geometry, this results in a time-dependent Aharonov-Casher phase (see Methods)

7 $${{\mathscr{A}}}_{\ell \ell ^{\prime} }(\tau )={\mu }_{m}{\int }_{{\overrightarrow{r}}_{\ell }}^{{\overrightarrow{r}}_{\ell ^{\prime} }}\overrightarrow{{\mathscr{A}}}(\tau )\cdot d\overrightarrow{\ell },$$

where ${\overrightarrow{r}}_{\ell }$ is the coordinate of the lattice at site $\ell$, and $\hslash =c=1$ has been used. We will use the magnonic Floquet-Bloch theory develop in Methods, and consider specific form of the vector potential.

A first choice would be a time-periodic vector potential in the *x*-*y* plane given by $$\overrightarrow{{\mathscr{A}}}(\tau )=[{{\mathscr{A}}}_{x}\,\sin (\omega \tau ),$$$${{\mathscr{A}}}_{y}\,\sin (\omega \tau +\varphi ),0]$$ with amplitudes $${{\mathscr{A}}}_{x}$$ and $${{\mathscr{A}}}_{y}$$. Here, *ϕ* = *π*/2 corresponds to circularly-polarized light and *ϕ* = 0 corresponds to linearly-polarized light. This form of vector potential is perpendicular to the DNLs and does not give any WPs^39,40^. In the magnonic honeycomb ferromagnetic system, the vector potential in the *x*-*y* plane gives rise to a photoinduced next-nearest-neighbour DM interaction in the *x*-*y* plane pointing along the *z*-direction. This term breaks $${\mathscr{T}}$$-symmetry, but yields a 3D magnonic Floquet TI similar to the 2D system^50^. Thus, there is no magnonic Floquet WPs for this choice of vector potential.

However, the 3D nature of the current model gives us another option for the vector potential. Now, we consider a different time-periodic vector potential in the *y*-*z* plane given by $\overrightarrow{{\mathscr{A}}}(\tau )=[0,{{\mathscr{A}}}_{y}\,\sin (\omega \tau ),{{\mathscr{A}}}_{z}\,\sin (\omega \tau +\varphi )]$ with amplitudes ${{\mathscr{A}}}_{y}$ and ${{\mathscr{A}}}_{z}$. This form of vector potential is parallel to the DNLs, hence WPs are expected to emerge^39,40^.

The time-dependent Hamiltonian ${\mathcal H} (\overrightarrow{k},\tau )$ is given by

8 $$ {\mathcal H} (\overrightarrow{k},\tau )={\rho }_{0}{1}_{2\times 2}+(\begin{array}{cc}0 & \rho (\overrightarrow{k},\tau )\\ {\rho }^{\ast }(\overrightarrow{k},\tau ) & 0\end{array}),$$

where $\rho (\overrightarrow{k},\tau )=\rho ({k}_{z},\tau )+\rho ({\overrightarrow{k}}_{\parallel },\tau )$, $\rho ({k}_{z},\tau )=-\,{t}_{L}{e}^{i({k}_{z}+\overrightarrow{{\mathscr{A}}}(\tau ))}$ and $\rho ({\overrightarrow{k}}_{\parallel },\tau )=-\,t{\sum }_{j}{e}^{i({\overrightarrow{k}}_{\parallel }+\overrightarrow{{\mathscr{A}}}(\tau ))\cdot {\overrightarrow{d}}_{j}}$. The corresponding Fourier components of the Hamiltonian (8) are given by

9 $${ {\mathcal H} }_{q}(\overrightarrow{k})={\rho }_{0}{1}_{2\times 2}+(\begin{array}{cc}0 & {\rho }_{q}(\overrightarrow{k})\\ {\rho }_{-q}^{\ast }(\overrightarrow{k}) & 0\end{array}).$$

For the vector potential in the *y*-*z* plane, we have

10 $${\rho }_{q}({k}_{z})=-\,{t}_{L}{{\mathscr{J}}}_{q}({{\mathscr{A}}}_{z}){e}^{i{k}_{z}}{e}^{iq\varphi },\,{\rho }_{q}({\overrightarrow{k}}_{\parallel })=-\,\sum _{j=1}^{3}{t}_{j,q}{e}^{i{\overrightarrow{k}}_{\parallel }\cdot {\overrightarrow{d}}_{j}},$$

where the renormalized interactions in this case are given by ${t}_{\mathrm{1,}q}=t{{\mathscr{J}}}_{-q}({{\mathscr{A}}}_{y}/2),\,{t}_{\mathrm{2,}q}=t{{\mathscr{J}}}_{-q}({{\mathscr{A}}}_{y}\mathrm{/2}),\,{t}_{3,q}=t{{\mathscr{J}}}_{q}({{\mathscr{A}}}_{y})$. Next, we study the high frequency regime ($\omega \gg {\rm{\Delta }}$), when the driving frequency *ω* is larger than the magnon bandwidth Δ. In this regime the Floquet sidebands are decoupled, and the system can be described by a time-independent effective Hamiltonian^28,30,31^, which can be obtained perturbatively in 1/*ω* expansion as

11 $${ {\mathcal H} }_{{\rm{eff}}}(\overrightarrow{k})={ {\mathcal H} }_{0}(\overrightarrow{k})-\frac{1}{\omega }([{ {\mathcal H} }_{0}(\overrightarrow{k}),{ {\mathcal H} }_{-1}(\overrightarrow{k})]-[{ {\mathcal H} }_{0}(\overrightarrow{k}),{ {\mathcal H} }_{1}(\overrightarrow{k})]+[{ {\mathcal H} }_{-1}(\overrightarrow{k}),{ {\mathcal H} }_{1}(\overrightarrow{k})]),$$

where ${ {\mathcal H} }_{0}(\overrightarrow{k})$ is the zeroth order Hamiltonian and ${ {\mathcal H} }_{\pm 1}(\overrightarrow{k})$ are the single photon dressed Hamiltonians. In the effective model expanded near the $\bar{{\rm{K}}}$-point, the first two commutators can be neglected. In the current system, however, we will not consider the effective model near the crossing point, and thus there is no reason to neglect the first two commutators since they can have a nonzero contribution away from the $\bar{{\rm{K}}}$-point.

**Figure 3:**
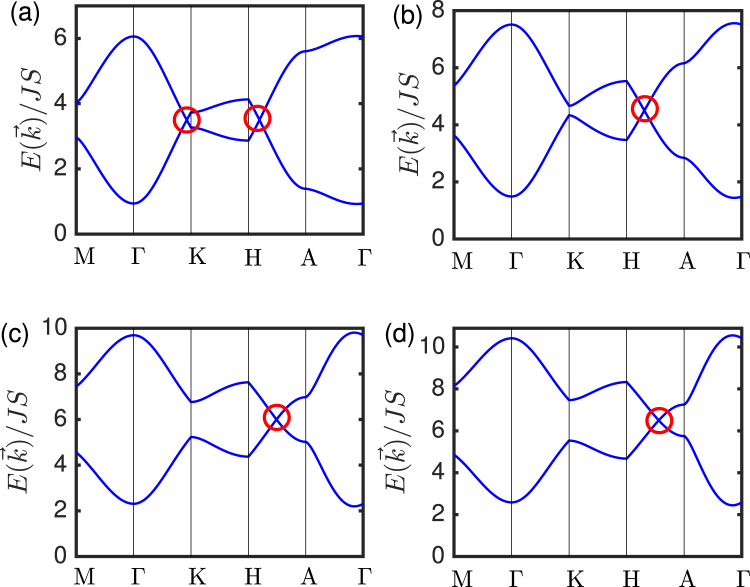
Magnon bands of periodically driven layered honeycomb ferromagnets for ${{\mathscr{A}}}_{z}={{\mathscr{A}}}_{y}=1.7,\,\varphi =\pi \mathrm{/2}$, and *ω*/*t* = 10. (**a**) *t*_*L*_/*t* = 0.5, (**b**) *t*_*L*_/*t* = 1.5, (**c**) *t*_*L*_/*t* = 3, (**d**) *t*_*L*_/*t* = 3.5. The red circle denotes the photoinduced magnonic WPs.

We have shown the effect of circularly-polarized light on the DNL magnons and the trivial magnon insulator in Fig. 3(a–d) respectively. For circularly-polarized light, *i.e*. $\varphi =\pi /2$, we find that the DNL magnons for $t/{t}_{L} < 3$ are not gapped out, but transform to photoinduced magnonic WPs as shown in Fig. 3(a–c). Interestingly, circularly-polarized light also closes the gap in the trivial magnon insulator phase for $t/{t}_{L} > 3$, thereby generating photoinduced magnonic WPs as shown in Fig. 3(d). Thus, both rotational and time-reversal symmetries are broken by photo-irradiation. We note that additional linear magnon band crossings occur along Γ–K line depending on the model parameters.

We have derived the expression for the effective Hamiltonian in Eq. (11) (see Methods). We find that the perturbative corrections to ${ {\mathcal H} }_{0}(\overrightarrow{k})$ gives a term proportional to ${f}_{z}(\overrightarrow{k}){\sigma }_{z}$ in the effective Hamiltonian (11). Indeed, this term breaks ${\mathscr{T}}$-symmetry (*i.e*. ${f}_{z}(-\,\overrightarrow{k})\ne {f}_{z}(\overrightarrow{k})$), and thus imposes an additional condition ${f}_{x}(\overrightarrow{k})=$ ${f}_{y}(\overrightarrow{k})={f}_{z}(\overrightarrow{k})=0$ for magnon band crossing to occur. The main result of this report is that photo-irradiation in the direction perpendicular to the DNLs generates 3D magnonic Floquet TIs, whereas photo-irradiation in the direction parallel to the DNLs generates 3D magnonic Floquet WPs, in analogy to electronic systems^39,40^. We would also like to mention that the time-periodic vector potential in the *y*-*z* plane does not generate a photo-induced next-nearest-neighbour DM interaction in the *x*-direction, since this term does not make any contribution to the magnon bands in linear spin wave theory. Therefore, the magnonic Floquet WPs in the current model do not originate from the out-of-plane DM interaction mechanism as opposed to magnonic WPs in the undriven 3D quantum ferromagnets^8–11^.

### Monopoles of the Berry curvatures

The band structures of the undriven DNL magnons in Fig. (2) are very similar to the corresponding photoinduced magnonic WPs in Fig. (3). This suggests that the analysis of the magnon band structures cannot sufficiently distinguish between DNLs and WPs. To distinguish the two, we need to compute the Berry curvature associated with the magnon band crossing points. As we noted above, the Berry curvature vanishes in the undriven DNLs as well as gapped trivial insulators as a result of ${\mathscr{T}}$ symmetry. Therefore, a non-vanishing Berry curvature with linear magnon band crossing must be a consequence of WPs due to broken ${\mathscr{T}}$ symmetry. In general, WPs are the source or sink of the Berry curvature, which means that a single WP can be considered as a monopole of the Berry curvature in momentum space.

**Figure 4:**
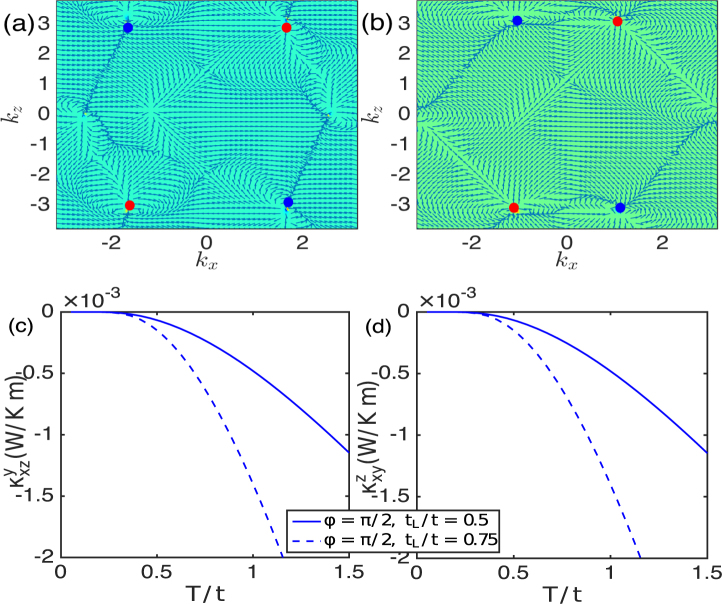
Top panel. Monopole distributions of the lowest magnon band Berry curvature ${{\rm{\Omega }}}_{\mathrm{1,}xz}^{y}(\overrightarrow{k})$ for the photoinduced magnonic WPs at (**a**) *t*_*L*_/*t* = 1.5 and (**b**) *t*_*L*_/*t* = 3.5. Bottom panel. The thermal Hall conductivity in units of *k*_*B*_. (**c**) ${\kappa }_{xz}^{y}$ vs. *T*/*t* and (**d**) ${\kappa }_{xy}^{z}$ vs. *T*/*t*. Here we set ${{\mathscr{A}}}_{z}={{\mathscr{A}}}_{y}=1.7$, *ϕ* = *π*/2, and *ω*/*t* = 10.

We define the Berry curvature of a given magnon band *n* as

12 $${{\rm{\Omega }}}_{n,ij}^{\ell }(\overrightarrow{k})=-\,\sum _{n^{\prime} \ne n}\frac{2{\rm{Im}}[\langle {\psi }_{n}(\overrightarrow{k})|{\hat{v}}_{i}|{\psi }_{n^{\prime} }(\overrightarrow{k})\rangle \langle {\psi }_{n^{\prime} }(\overrightarrow{k})|{\hat{v}}_{j}|{\psi }_{n}(\overrightarrow{k})\rangle ]}{{[{\epsilon }_{n}(\overrightarrow{k})-{\epsilon }_{n^{\prime} }(\overrightarrow{k})]}^{2}},$$

where ${\hat{v}}_{i}=\partial { {\mathcal H} }_{{\rm{eff}}}(\overrightarrow{k})/\partial {k}_{i}$ are the velocity operators, ${\psi }_{n}(\overrightarrow{k})$ are the magnon eigenvectors, and ${\varepsilon }_{\alpha }(\overrightarrow{k})$ are the magnon quasi-energies. The Berry curvature can be considered as a pseudo-vector pointing along the $\ell$ directions perpendicular to both the *i* and *j* directions. All the components of the Berry curvature are found to be nonzero. In the top panel of Fig. (4), we have shown the plot of the monopole field distributions of the lowest magnon band Berry curvature ${{\rm{\Omega }}}_{\alpha ,xz}^{y}(\overrightarrow{k})$ (with *α* = 1) in the *k*_*y*_ = 0 plane. We note that the Berry curvature is maximized at the photoinduced magnonic WPs. The monopole distribution of the Berry curvature is a compelling evidence that the photoinduced magnon band crossings are indeed magnonic WPs.

### Anomalous thermal Hall effect

In analogy to anomalous Hall effect in electronic Weyl semimetals^73,74^, the magnonic WPs in 3D quantum magnets also exhibit the anomalous thermal Hall effect^12^, which is generally understood as a consequence of the nonvanshing Berry curvatures. In the high frequency limit, the system is close to equilibrium. Thus, the same theoretical concept of undriven anomalous thermal Hall effect due to a temperature gradient^75,76^ is applicable to the driven system close to thermal equilibrium. The transverse components ${\kappa }_{ij}^{\ell }$ of the thermal Hall conductivity are given by^76,77^

13 $${\kappa }_{ij}^{\ell }=-\,{k}_{B}^{2}T{\int }_{BZ}\frac{{d}^{3}k}{{\mathrm{(2}\pi )}^{3}}\sum _{n=1}^{N}{c}_{2}({f}_{n}^{B}){{\rm{\Omega }}}_{n,ij}^{\ell }(\overrightarrow{k}).$$

Here, ${f}_{n}^{B}=1/({e}^{{\varepsilon }_{n}(\overrightarrow{k})/{k}_{B}T}-1)$ is the Bose distribution function close to thermal equilibrium, *k*_*B*_ is the Boltzmann constant, *T* is the temperature, and ${c}_{2}(x)=(1+x){(\mathrm{ln}\frac{1+x}{x})}^{2}-{(\mathrm{ln}x)}^{2}-2{{\rm{Li}}}_{2}(\,-\,x)$, with Li_2_(*x*) being the dilogarithm. Similar to the Berry curvature, ${\kappa }_{ij}^{\ell }$ vanishes in the undriven DNLs and gapped trivial insulators due to ${\mathscr{T}}$-symmetry. The maximum contribution to ${\kappa }_{ij}^{\ell }$ comes from the photoinduced magnonic WPs at the lowest magnon excitation due to the Berry curvature. It can be shown that ${\kappa }_{ij}^{\ell }$ depends on the distribution of magnonic WPs in momentum space^12^, in analogy to the thermal Hall effect in electronic Weyl semimetals^78^. In the bottom panel of Fig. (4), we have shown the trends of (c) ${\kappa }_{xz}^{y}$ and (d) ${\kappa }_{xy}^{z}$ in the photoinduced Weyl magnon phase.

## Conclusion

The main result of this report is that magnonic WPs can be photoinduced in three-dimensional (3D) quantum magnets initially possessing DNL magnon and gapped trivial magnon insulator phases. We achieved this result by utilizing magnons as hopping magnetic dipole moment in an ordered quantum magnet. Hence, magnons couple to time-dependent electric field through the time-dependent Aharonov-Casher effect as shown in Methods. In other words, the electric charge in electronic systems is dual to the magnetic dipole moment in magnonic systems. The newly proposed magnonic Floquet WPs have many advantages over intrinsic magnonic WPs. First, they can be tuned by the incident light, and can also be engineered in different magnetic systems. Second, they do not rely on intrinsic perturbative interactions to break time-reversal symmetry, and they could also provide a platform for investigating new features such as photo-magnonics^55^, magnon spintronics^56,57^, and ultrafast optical control of magnetic spin currents^58–61^. Therefore, the current results are also pertinent to experimental investigation, and can be applied to different bulk 3D quantum magnetic materials. Thereby, extending the notion of magnonic WPs to a broader class of 3D quantum magnets.

We note that there is very little spectroscopic experimental progress in the observation of magnonic analogs of electronic topological systems. Recently, bulk Dirac magnons have been experimentally confirmed in the 3D antiferromagnet Cu_3_ TeO_6_^52–54^. The measurement of the anomalous thermal Hall effect^79–81^ is also an alternative way to confirm the existence of topological spin excitations in quantum magnets. It should be noted that the thermal Hall effect is absent in the undriven Dirac and nodal-line magnons, as well as the trivial magnon insulators, because of the presence of time-reversal-symmetry. Moreover, the chiral magnon edge and surface magnon modes are yet to be verified experimentally in topological magnon systems. In this report, we have focused on features that can be directly measured experimentally e.g. by using ultrafast terahertz spectroscopy, inelastic neutron scattering, and thermal Hall measurements.

## Methods

### Magnonic Floquet-Bloch theory

The Floquet-Bloch theory is a formalism for studying periodically driven quantum systems and it applies to different cases of physical interests. The magnonic version describes the interaction of light with magnonic Bloch states in insulating quantum magnets. In the present case, the time-dependent Hamiltonian ${\mathcal H} (\overrightarrow{k},\tau )$ can be obtained by making the time-dependent Peierls substitution $\overrightarrow{k}\to \overrightarrow{k}+\overrightarrow{{\mathscr{A}}}(\tau )$. Note that ${\mathcal H} (\overrightarrow{k},\tau )$ is periodic due to the time-periodicity of the vector potential.

Hence, it can be expanded in Fourier space as ${\mathcal H} (\overrightarrow{k},\tau )= {\mathcal H} (\overrightarrow{k},\tau +T)={\sum }_{n=-\infty }^{\infty }{e}^{in\omega \tau }{ {\mathcal H} }_{n}(\overrightarrow{k}),$ where ${ {\mathcal H} }_{n}(\overrightarrow{k})=\frac{1}{T}{\int }_{0}^{T}{e}^{-in\omega \tau } {\mathcal H} (\overrightarrow{k},\tau )d\tau ={ {\mathcal H} }_{-n}^{\dagger }(\overrightarrow{k})$ is the Fourier component. Thus, we can write its eigenvectors in the Floquet-Bloch form $|{\psi }_{\alpha }(\overrightarrow{k},\tau )\rangle ={e}^{-i{\epsilon }_{\alpha }(\overrightarrow{k})\tau }|{\xi }_{\alpha }(\overrightarrow{k},\tau )\rangle$, where $|{\xi }_{\alpha }(\overrightarrow{k},\tau )\rangle =|{\xi }_{\alpha }(\overrightarrow{k},\tau +T)\rangle ={\sum }_{n}{e}^{in\omega \tau }|{\xi }_{\alpha }^{n}(\overrightarrow{k})\rangle$ is the time-periodic Floquet-Bloch wave function of magnons and *ε*_*α*_$(\overrightarrow{k})$ are the magnon quasi-energies. We define the Floquet operator as ${ {\mathcal H} }^{F}(\overrightarrow{k},\tau )= {\mathcal H} (\overrightarrow{k},\tau )-i{\partial }_{\tau }$, which leads to the Floquet eigenvalue equation

14 $$\sum _{m}[{ {\mathcal H} }^{n-m}(\overrightarrow{k})+m\omega {\delta }_{n,m}]{\xi }_{\alpha }^{m}(\overrightarrow{k})={\varepsilon }_{\alpha }(\overrightarrow{k}){\xi }_{\alpha }^{n}(\overrightarrow{k}).$$

### Massless neutral particle in an external electromagnetic field

In this section, we give the general theory for a massless neutral particle with magnetic dipole moment such as the magnonic DNL quasiparticle, coupled non-minimally to an external electromagnetic field (denoted by the tensor *F*_*μν*_) via its magnetic dipole moment (*μ*). In (3 + 1) dimensions, the system is described by the Dirac-Pauli Lagrangian^82^

15 $$ {\mathcal L} =\bar{\psi }(x)(i{\gamma }^{\mu }{\partial }_{\mu }-\frac{\mu }{2}{\sigma }^{\mu \nu }{F}_{\mu \nu })\psi (x),$$

where $\hslash =c=1$ has been used. Here $x\equiv {x}^{\mu }=({x}^{0},\overrightarrow{x})$, $\bar{\psi }(x)={\psi }^{\dagger }(x){\gamma }^{0}$, and ${\gamma }^{\mu }=({\gamma }^{0},\overrightarrow{\gamma })$ are the 4 × 4 Dirac matrices that obey the algebra

16 $$\{{\gamma }^{\mu },{\gamma }^{\nu }\}=2{g}^{\mu \nu },\,{\rm{where}}\,{g}^{\mu \nu }={\rm{diag}}(1,-\,1,-\,1,-\,1),$$

and

17 $${\sigma }^{\mu \nu }=\frac{i}{2}[{\gamma }^{\mu },{\gamma }^{\nu }]=i{\gamma }^{\mu }{\gamma }^{\nu },\,(\mu \ne \nu ).$$

In this report, we will consider the system with only spatially uniform and time-varying electric field $\overrightarrow{ {\mathcal E} }(\tau )$. In this case, the corresponding Hamiltonian is given by

18 $$ {\mathcal H} =\int {d}^{3}x\,{\psi }^{\dagger }(x)[\overrightarrow{\alpha }\cdot (-\,i\overrightarrow{\nabla }-i\mu \beta \overrightarrow{ {\mathcal E} }(\tau ))]\psi (x),$$

where $$\overrightarrow{\alpha }={\gamma }^{0}\overrightarrow{\gamma }$$ and *β* = *γ*^0^.

In (2 + 1) dimensions, the Dirac matrices are simply Pauli matrices given by

19 $$\beta ={\gamma }^{0}={\sigma }_{z},\,{\gamma }^{1}=i{\sigma }_{y},\,{\gamma }^{2}=-\,i{\sigma }_{x}.$$

The corresponding momentum space Hamiltonian in (2 + 1) dimensions now takes the form

20 $$ {\mathcal H} =\int \frac{{d}^{2}k}{{(2\pi )}^{2}}\,{\psi }^{\dagger }(\overrightarrow{k},\tau ) {\mathcal H} (\overrightarrow{k},\tau )\psi (\overrightarrow{k},\tau ),$$

where

21 $$ {\mathcal H} (\overrightarrow{k},\tau )=\overrightarrow{\sigma }\cdot [\overrightarrow{k}+{\mu }_{m}(\overrightarrow{ {\mathcal E} }(\tau )\times \hat{z})],\,{\rm{with}}\,\overrightarrow{\sigma }=({\sigma }_{x},{\sigma }_{y}).$$

We see that the Hamiltonian in Eq. (21) is equivalent to that of DNL Hamiltonian in Eq. (3) near the crossing point, coupled to a time-periodic electric field through the magnetic dipole moment ${\overrightarrow{\mu }}_{m}={\mu }_{m}\hat{z}$, where *μ*_*m*_ = *gμ*_*B*_. The time-dependent Aharonov-Casher phase is evident from the Hamiltonian in Eq. (21). Due to the relation $\overrightarrow{ {\mathcal E} }(\tau )=-\,\partial \overrightarrow{{\mathscr{A}}}(\tau )/\partial \tau$, we can replace $\overrightarrow{ {\mathcal E} }(\tau )\times \hat{z}$ with $\overrightarrow{{\mathscr{A}}}(\tau )$ as in the main text. Hence, we write the Peierls substitution as $\overrightarrow{k}\to \overrightarrow{k}+\overrightarrow{{\mathscr{A}}}(\tau )$. We note that this replacement does not change our results, because we could also redefine the time-periodic electric field $\overrightarrow{ {\mathcal E} }(\tau )$ such that $\overrightarrow{ {\mathcal E} }(\tau )\times \hat{z}=[0,{ {\mathcal E} }_{y}\,\sin (\omega \tau ),{ {\mathcal E} }_{z}\,\sin (\omega \tau +\varphi )]$, where ${ {\mathcal E} }_{y,z}\equiv {{\mathscr{A}}}_{y,z}$.

### Effective Hamiltonian

In this section, we derive the form of the effective Hamiltonian in Eq. (11) in the case of a vector potential in the *y*-*z* plane. The effective Hamiltonian can be written as

22 $${ {\mathcal H} }_{{\rm{eff}}}(\overrightarrow{k})={f}_{0}{\sigma }_{0}+{f}_{x}^{0}(\overrightarrow{k}){\sigma }_{x}+{f}_{y}^{0}(\overrightarrow{k}){\sigma }_{y}+{f}_{z}(\overrightarrow{k}){\sigma }_{z},$$

where,

23 $${f}_{x}^{0}(\overrightarrow{k})=-\,\sum _{j=1}^{3}{t}_{j}^{0}\,\cos ({\overrightarrow{k}}_{\parallel }\cdot {\overrightarrow{d}}_{j})-{t}_{L}^{0}\,\cos ({k}_{z}),\,{f}_{y}^{0}(\overrightarrow{k})=\sum _{j=1}^{3}{t}_{j}^{0}\,\sin ({\overrightarrow{k}}_{\parallel }\cdot {\overrightarrow{d}}_{j})+{t}_{L}^{0}\,\sin ({k}_{z}),$$

with $${t}_{1}^{0}=t{{\mathscr{J}}}_{0}({{\mathscr{A}}}_{y}/2),\,{t}_{2}^{0}=t{{\mathscr{J}}}_{0}({{\mathscr{A}}}_{y}\mathrm{/2}),\,{t}_{3}^{0}=t{{\mathscr{J}}}_{0}({{\mathscr{A}}}_{y})$$, and $${t}_{L}^{0}={t}_{L}{{\mathscr{J}}}_{0}({{\mathscr{A}}}_{z})$$.

24 $$\begin{array}{lll}{f}_{z}(\overrightarrow{k}) & = & \frac{4}{\omega }[2{t}^{2}{{\mathscr{J}}}_{0}({{\mathscr{A}}}_{y}\mathrm{/2}){{\mathscr{J}}}_{1}({{\mathscr{A}}}_{y}\mathrm{/2})+{t}^{2}{{\mathscr{J}}}_{0}({{\mathscr{A}}}_{y}){{\mathscr{J}}}_{1}({{\mathscr{A}}}_{y})+2t{t}_{L}{{\mathscr{J}}}_{0}({{\mathscr{A}}}_{z}){{\mathscr{J}}}_{1}({{\mathscr{A}}}_{y})\cos (\sqrt{3}{k}_{x})\\  &  & +t{t}_{L}{{\mathscr{J}}}_{0}({{\mathscr{A}}}_{z}){{\mathscr{J}}}_{1}({{\mathscr{A}}}_{y})\cos ({k}_{y}-{k}_{z})+{{\mathscr{J}}}_{1}({{\mathscr{A}}}_{z})\{{t}_{L}^{2}{{\mathscr{J}}}_{0}({{\mathscr{A}}}_{z})+t{t}_{L}{{\mathscr{J}}}_{0}({{\mathscr{A}}}_{y})\cos ({k}_{y}-{k}_{z})\}\,\cos (\varphi )\\  &  & +2\,\cos (\sqrt{3}{k}_{x}/2)[{t}^{2}\{{{\mathscr{J}}}_{0}({{\mathscr{A}}}_{y}){{\mathscr{J}}}_{1}({{\mathscr{A}}}_{y}\mathrm{/2})+{{\mathscr{J}}}_{0}({{\mathscr{A}}}_{y}\mathrm{/2}){{\mathscr{J}}}_{1}({{\mathscr{A}}}_{y})\}\,\cos (\sqrt{3}{k}_{y}/2)\\  &  & +t{t}_{L}\{{{\mathscr{J}}}_{0}({{\mathscr{A}}}_{z}){{\mathscr{J}}}_{1}({{\mathscr{A}}}_{y}/2)+{{\mathscr{J}}}_{0}({{\mathscr{A}}}_{y}\mathrm{/2}){{\mathscr{J}}}_{1}({{\mathscr{A}}}_{z})\cos (\varphi )\}\,\cos (\sqrt{3}{k}_{y}\mathrm{/2}+{k}_{z})]\\  &  & -t{t}_{L}{{\mathscr{J}}}_{1}({{\mathscr{A}}}_{z})\sin (\varphi )\{{{\mathscr{J}}}_{1}({{\mathscr{A}}}_{y})\sin ({k}_{y}-{k}_{z})-2{{\mathscr{J}}}_{1}({{\mathscr{A}}}_{y}/2)\cos (\sqrt{3}{k}_{x}\mathrm{/2})\sin ({k}_{y}\mathrm{/2}+{k}_{z})\}].\end{array}$$
